# Correspondence

**Published:** 1883-11

**Authors:** 


### Correspondence.

Among the many letters which the Committee on Legislation of the Erie County Medical Society have received from prominent medical men in the State, cordially endorsing the proposed bill, we have been permitted to print one from Dr. Moore, which will be read with no little interest:

ROCHESTER, October 22, 1883.

DEAR DOCTOR—I have not been unmindful of the matter your committee have in hand, having been a sort of pioneer in the work. When I got my committee of the State Medical Society together ten years ago, and laid my plans before them to get a board in operation under the Regents, in hopes some day of enlarging their powers, I literally stood alone. But the law was passed, and I waited long for the fruits. It has now taken a new turn. While I have an instinctive dislike of any association with outsiders, it must be confessed that the plan your committee have in hand is the only one that can insure uniformity. Moreover, it is impossible to dictate anything on the subject of therapeutics. This, perhaps, is no great calamity if men are thoroughly informed in the other departments of medical knowledge.

If it is customary, and of this I cannot speak, to make a preamble to an act, I think this should be done, to show that the object is to strike at ignorance and not at any therapeutic faith.

Yours truly,

E. M. MOORE.

---

LYONS, October 18, 1883.

*Editors Buffalo Medical and Surgical Journal:*

DEAR DOCTORS—At the regular meeting of the Wayne County Medical Society, held on the 9th inst., a copy of the proposed bill for the purpose of establishing a State Medical Faculty, adopted by the Erie County Medical Society, was presented for consideration. Upon motion, it was—

*Resolved*, That we heartily endorse the bill proposed by the Erie County Medical Society, and that we use all lawful means to further its passage.

*Resolved*, That a committee of one in each assembly district be appointed to present the bill to our candidates for the assembly.

The President appointed as such committees: Eastern district, Dr. Darwin Colvin, of Clyde; Western district, Dr. C. G. Pomeroy, of Newark.

Very respectfully,

FLETCHER J. SHERMAN,

Secretary.

---

WHITE PLAINS, N. Y., Oct. 20, 1883.

*Editors Buffalo Medical and Surgical Journal:*

DEAR SIRS—Yours of the 18th is at hand. I presented the communication from Erie County Medical Society at the last meeting of Westchester County Medical Society, and also read the proposed bill which expresses in detail what was embraced in the resolutions of the society, passed at its annual meeting. The bill was endorsed by the society, and its strongest influence will be exerted to secure its passage. The President appointed Drs. Geo. J. Fisher and Wm. H. Helm as a committee to urge the passage of the bill.

Yours truly,

N. F. CURTIS,

Secretary.

---

The following circular, received by the Secretary of the Erie County Society, since its last meeting, gives the resolution and action of the society above referred to:

WHITE PLAINS, N. Y., Sept. 26, 1883.

*Dr. A. M. Barker, Secretary of the Erie Co. Medical Society:*

At the annual meeting of the Medical Society of the county of Westchester, in June last, the Board of Censors presented the following resolutions, which were adopted:

*Resolved*, That the representatives of this county in the State legislature be requested to urge the passage of an act to prohibit all medical colleges from granting diplomas, and requesting all candidates for the degree of Doctor in Medicine to appear before a State Board of Medical Examiners who shall recommend successful candidates to the Regents of the State University for a diploma.

*Resolved*, That the Secretary send a copy of this resolution to the members of the legislature from this county, and to every County Medical Society in the State, and urge them to take the same action.

We think this resolution expresses the universal sentiment of a profession crowded with those who have virtually bought their diplomas. Let us earnestly co-operate to obtain the desired legislation. Please inform me of the action of your society.

N. F. CURTIS, Secretary.

